# School Readiness Among Children Insured by Medicaid, South Carolina

**DOI:** 10.5888/pcd9.110333

**Published:** 2012-06-07

**Authors:** William B. Pittard, Thomas C. Hulsey, James N. Laditka, Sarah B. Laditka

**Affiliations:** Author Affiliations: Thomas C. Hulsey, Medical University of South Carolina, Charleston, South Carolina; James N. Laditka, Sarah B. Laditka, University of North Carolina at Charlotte, Charlotte, North Carolina.

## Abstract

**Introduction:**

The American Academy of Pediatrics recommends a schedule of age-specific well-child visits through age 21 years. For children insured by Medicaid, these visits are called Early and Periodic Screening, Diagnosis, and Treatment (EPSDT). These visits are designed to promote physical, emotional, and cognitive health. Six visits are recommended for the first year of life, 3 for the second year. We hypothesized that children with the recommended visits in the first 2 years of life would be more likely than others to be ready for school when they finish kindergarten.

**Methods:**

We studied children insured by Medicaid in South Carolina, born during 2000 through 2002 (n = 21,998). Measures included the number of EPSDT visits in the first 2 years of life and an assessment of school readiness conducted at the end of kindergarten. We used logistic regression to examine the adjusted association between having the recommended visits and school readiness, controlling for characteristics of mothers, infants, prenatal care and delivery, and residence area.

**Results:**

Children with the recommended visits had 23% higher adjusted odds of being ready for school than those with fewer visits.

**Conclusion:**

EPSDT may contribute to school readiness for children insured by Medicaid. Children having fewer than the recommended EPSDT visits may benefit from school readiness programs.

## Introduction

Lack of school readiness is a public health concern with adverse physical, psychological, social, and economic consequences for many children, particularly those insured by Medicaid (1,2). Children unprepared for school often perform poorly academically, have low self-esteem, and in the long term are at greater risk than others for unemployment, poverty, and crime (3,4). School readiness begins to develop early in life, well before formal schooling (5-9). Inadequate school readiness has been associated with poverty and poor health (10-12), a lack of reading materials and cognitive stimulation in the home, and cultural variation in beliefs and attitudes about education (2,3).

Well-child care may contribute to school readiness (13). In Medicaid, well-child care is called Early and Periodic Screening, Diagnosis, and Treatment (EPSDT). The American Academy of Pediatrics (AAP) recommends a widely accepted national standard of well-child care, including at least 6 EPSDT visits in the first year of life and at least 3 in the second (14). The recommended content for EPSDT visits in the South Carolina Medicaid system is all age-based preventive care services recommended by the AAP, screening procedures designed to promote normal child development and school readiness, and anticipatory guidance for parents or caregivers (5,11,15-17). Anticipatory guidance during a well-child visit gives parents education and counseling intended to promote child health. For example, these visits include advice about physical activity, nutrition, appropriate use of health care, parent–child reading, and avoiding exposure to household toxins such as lead.

The AAP has called for research on the effectiveness of EPSDT. However, this effectiveness has been confirmed only for vaccinations (18). A large proportion of children do not have the recommended number of EPSDT visits (19,20). Underuse of these visits and lack of readiness for first-grade learning disproportionately affect children insured by Medicaid (1,3,13).

EPSDT visits provide an opportunity for clinicians to identify and address physical, developmental, emotional, social, or other problems that may impede optimal development. Beginning with anticipatory guidance during prenatal care, the visits include vaccinations, developmental and sensory evaluations, evaluation of nutrition and oral health, guidance about parenting, and other preventive services. Parents of children with the recommended number of EPSDT visits in infancy also receive more information than others about cognitive stimulation for their children and about avoiding risks to cognitive health such as lead exposure, accidents, and undernutrition (8,11,15). AAP guidelines promote guidance during these visits about excessive television watching, which has been associated with attention deficit hyperactivity disorder, poor school performance, and possible delays in the development of language skills among infants younger than 2 years (21). Thus, our hypothesis is that children insured by Medicaid who have at least the AAP-recommended number of EPSDT visits in the first 2 years of life will be more likely to be ready for school when they finish kindergarten than those with fewer visits.

## Methods

### Study design

The data represent South Carolina children born during 2000 through 2002 and consistently enrolled in Medicaid in the first 2 years of life. Data were from linked state Medicaid claims, birth certificate records, and state Department of Education kindergarten school readiness assessments. The study was approved by the institutional review board of the Medical University of South Carolina and the South Carolina Data Oversight Commission. Previous research using South Carolina Medicaid data suggests that the data and linkages required for this research have a high degree of validity and completeness (22,23).

### Outcome measure

The dependent variable represents results of the South Carolina Readiness Assessment (SCRA). Kindergarten teachers throughout the state conduct these school readiness assessments at the end of the kindergarten (K-5) school year (24). The SCRA assesses how well students meet state school readiness standards for personal and social development, English language arts, and mathematics (25) (Appendix). Readiness is defined using guidelines from the National Education Goals Panel (26,27). Recommended EPSDT content addresses the cognitive, emotional, physical, and social development needed to perform adequately in these assessments (13,21). Evidence indicates that results from the SCRA predict success in school (28).

### Exposure variable

The independent variable of primary interest was whether each child had the recommended number of EPSDT visits in the first 2 years of life. These visits could be for vaccinations, screening services, and parental anticipatory guidance. They were identified using claims submitted by providers, which were categorized when received by Medicaid using codes developed for the Medicaid system (H. Kirby, Office of Research and Statistics, South Carolina Budget and Control Board, written communication).

### Control variables

Control variables included several characteristics of mothers: age in years at the child’s delivery; years of education; marital status; race/ethnicity; rural or urban residence; parity; whether the delivery was vaginal; and whether the mother received adequate prenatal care, as defined by the Kessner Adequacy of Prenatal Care Index (29). Family income was characterized as either 1) less than or equal to 50% of the income level defined by federal guidelines as poverty or 2) greater than 50% to 185% of poverty (which included all other families in SC Medicaid). Controls for child characteristics include birth weight in grams, sex, and gestational age in weeks.

To control for baseline health status, we excluded children with conditions that might predispose them to have more health provider visits, which could result in more visits being coded as EPSDT visits. Such conditions also affect other parenting behaviors and could be associated with school readiness (30). Thus, we excluded children with birth admissions lasting 7 days or more; major heart diseases or conditions; central nervous system malformations; recognizable genetic malformations; and anomalies including genitourinary, gastrointestinal, and musculoskeletal (identified using *International Classification of Diseases, Ninth Revision, Clinical Modification* codes). Study children were also restricted to those who were full term with appropriate weight for gestational age by requiring 37 to 42 weeks’ gestational age and by excluding children with birth weights outside published fetal growth norms (31). Children are often not enrolled in their Medicaid insurance plan until sometime during the first month of life. Thus, we calculated the number of visits for the first year based on the 2nd through the 12th months of life.

### Study population

In 2000 through 2002, there were 61,112 South Carolina births insured by Medicaid. The study restrictions excluded 16,280 (26.6%), leaving 44,832. Most exclusions were due to birth anomalies (n = 11,552), birth weight outside fetal growth norms (n = 6,878), and gestational age less than 37 or greater than 42 weeks (n = 7,491). Of the children excluded, 5,913 met more than 1 of these criteria. Of the remaining cohort, 42,485 (94.8%) met the requirements of being enrolled in Medicaid throughout the first 2 years of life and having a successful linkage of their Medicaid claims and birth certificate files. The final number of study children was established with successful SCRA data linkage for 31,751 (74.7%) of these 42,485 children.

Of those 31,751 children, 9,753 (30.7%) had missing values for at least 1 variable used in the analysis. These observations were excluded from the multivariate analysis, leaving a final analytical sample of 21,998. We conducted a separate analysis to examine the sensitivity of our results to this exclusion of observations with missing values. For that analysis, we included a dummy variable in the model associated with each variable having a missing value, indicating this missing data. Doing so permitted an estimation of the models using all 31,751 observations. The result of this sensitivity analysis was consistent with the results we report, although indicating an even larger association between EPSDT and school readiness. Thus, we report conservative results for this relationship.

### Analytic approach

Each of the 14 SCRA domain evaluations was scored on a scale of 1 to 3 (1 = rarely or never demonstrates the assessed characteristic, 2 = sometimes demonstrates, and 3 = consistently demonstrates). Of the 21,998 children, 64.6% received “consistently demonstrates” for at least 10 of the 14 evaluations and were considered ready for first grade; 35.4% received “consistently demonstrates” for fewer than 10 domains and were considered not ready for first grade. The cut-point that defined these categories is the official measure used by the South Carolina Department of Education (A. Brailsford, Coordinator of Development, South Carolina Department of Education, oral communication).

Bivariate analyses compared characteristics of mothers and children in 2 groups: children who had the recommended number of EPSDT visits and those who had fewer visits (χ^2^ for categorical variables; *t* tests for continuous variables). The χ^2^ test also assessed the association between having the recommended number of EPSDT visits and later school readiness. Multivariate analyses using logistic regression estimated the relative odds of the same association, adjusted for the maternal and child characteristics examined.

## Results

Mothers of children who had the AAP-recommended number of EPSDT visits in both years had slightly more education than others (11.9 y vs 11.6 y, *P* < .001) (Table). They were also more likely to be married, nulliparous, white, urban residents, and to have both income greater than 50% of the income level defined as poverty and adequate prenatal care. The analogous bivariate comparisons of maternal and child characteristics associated with having the recommended number of EPSDT visits in the first year of life, and separately for the second year, suggested the same relationship for each of the 2 years (data not shown).

**Table:** Maternal and Child Characteristics for Medicaid-Insured Children Born During 2000–2002 and the Number of Early and Periodic Screening, Diagnosis, and Treatment (EPSDT) Visits During the First and Second Years of Life, South Carolina^a^

Characteristic	Had at Least the Recommended^b^ No. of EPSDT Visits		*P* Value^c^
	Yes	No	
**Mothers’ characteristics**			
**Age at delivery, mean (SD), y**	23.0 (5.3)	23.1 (5.2)	.43
**Education, mean (SD), y**	11.9 (1.8)	11.6 (1.9)	< .001
**Married, n (%)**	483 (26.4)	4,505 (22.3)	< .001
**Nulliparous, n (%)**	1,027 (56.2)	8,129 (40.3)	< .001
**Vaginal delivery, n (%)**	1,571 (85.9)	17,606 (87.3)	.099
**Race/ethnicity, n (%)**			
White	821 (44.9)	6,946 (34.4)	< .001
African American	927 (50.7)	12,056 (59.8)	< .001
Hispanic	53 (2.9)	877 (4.4)	< .003
Other	27 (1.5)	291 (1.4)	.91
**Urban residence, n (%)**	1,292 (70.7)	12,509 (62.0)	< .001
**Family income ≤50% of federal poverty guidelines, n (%)**	378 (20.7)	5,439 (27.0)	< .001
**Adequate prenatal care, n (%)^d^**	1,274 (69.7)	12,642 (62.7)	< .001
**Children’s characteristics**			
**Male, n (%)**	876 (47.9)	9,769 (48.4)	.68
**Gestational age, mean (SD), weeks**	39.1 (1.2)	39.1 (1.1)	.18
**Birth weight, mean (SD), g**	3,258 (401)	3,258 (398)	.95
**Had recommended no. of EPSDT visits**			
First year of life, n (%)	2,455 (11.2)	19,543 (88.8)	< .001
Second year of life, n (%)	5,893 (26.8)	16,105 (73.2)	< .001
First and second years combined, n (%)	1,828 (8.3)	20,170 (91.7)	< .001

^a^ Data source: South Carolina Office of Research and Statistics, representing children enrolled in Medicaid from birth until second birthday, linked with their South Carolina Readiness Assessment of preparedness for school (n = 21,998).

^b^ As recommended by the American Academy of Pediatrics (14).

^c^ Calculated by using χ^2^ tests for categorical variables, *t* tests for continuous variables.

^d^ Identifies mothers who had at least adequate prenatal care as defined by the Kessner Adequacy of Prenatal Care Index (29).

Only 11.2% and 26.8% of children, respectively, had at least the recommended number of EPSDT visits in their first and second years (Table). Approximately 8% of children had the recommended number of EPSDT visits in both their first and second years. Children having fewer than the recommended number of EPSDT visits had mean (standard deviation) visit rates of 3.1 (1.7) in their first year, 1.0 (0.8) in their second year, and 4.7 (2.5) for their first and second year combined.

In the analysis focused on the first year of life, 68.9% of children with at least the recommended number of visits were ready for school, compared with 64.1% of those with fewer visits (*P* < .001). The corresponding percentages associated with EPSDT visits in the second year of life were 68.6% and 63.2% (*P* < .001). For their first and second year combined, 71.0% of children with at least the AAP-recommended number of visits were ready for school, compared with 64.1% of those with fewer visits (*P* < .001). The adjusted odds of being ready for school were greater for those with the recommended number of EPSDT visits in the first year of life compared with those having fewer visits (odds ratio [OR], 1.12; 95% confidence interval [CI], 1.02–1.23). The corresponding adjusted OR associated with EPSDT visits in the second year of life was 1.19 (95% CI, 1.10–1.26). For children with the recommended number of EPSDT visits in their first and second year, the adjusted odds of being ready for school were greater than for those with fewer visits (OR, 1.23; 95% CI, 1.10–1.37).

Approximately 75% of the observations representing children with Medicaid records for EPSDT visits in infancy could be linked with education data for the SCRA. To examine whether this linkage rate might have biased the results, we compared maternal and child characteristics of the children linked with their SCRA evaluations with those not successfully linked. Those linked were more likely to be African American (54.9% vs 45.7%, *P* < .001), their mothers were less likely to be married (22.7% vs 27.9%, *P* < .001), and they were less likely to be Hispanic (6.1% vs 9.0%, *P* < .001). There were no clinically significant differences between the 2 groups in the mean number of EPSDT visits or in the proportions that had at least the AAP-recommended EPSDT visits in their first year, their second year, or their first and second year combined.

## Discussion

In this study of children insured by Medicaid in South Carolina, consistent with our hypothesis, children having at least the AAP-recommended number of EPSDT visits in the first 2 years of life were more likely to be ready for school at the end of the K–5 school year. This finding is consistent with the expectation that mothers of children having at least the recommended number of EPSDT visits in the first 2 years of life receive more guidance about child health and development and parenting and that their children receive more screening and preventive care (5,6,9,13). Thus, for example, children with the recommended EPSDT visits may be less likely to have unaddressed vision or hearing impairments, which can affect both cognitive and social development as well as performance in kindergarten. Parents whose children have the recommended number of visits receive regular health education and guidance about risk avoidance, cognitive development and emotional health, and social development. As a result, they may improve their children’s diets, promote physical activity, arrange more social activity, and avoid environmental toxins, all of which may be associated with cognitive development and the social skills that are evaluated in the school readiness assessment (8,13,16,17).

The clinical effectiveness of well-child care other than vaccinations has not been objectively confirmed. State Medicaid program administrators often question whether increasing well-child care in infancy is worth the added cost (18,32). Yet lack of school readiness has long-term consequences for children and society. The study represented infants born to mothers insured by Medicaid. Families insured by Medicaid often have limited access to health care even though they are insured. They are more likely to have problems accessing needed services due to factors such as limited health literacy, difficulty arranging for child care or time away from employment, and problems getting transportation to obtain needed services. Infants in these families are at high risk of poor health and poor social outcomes. The difference we found in school readiness associated with EPSDT may indicate an opportunity to improve infant outcomes by expanding use of EPSDT services. In our analysis, approximately 8% of children had the recommended number of EPSDT visits in the first 2 years of life. The potential to increase this percentage, and possibly the rate of readiness for school, is large. Improving school readiness by correcting underuse of well-child care by children insured by Medicaid (92% did not have the recommended number of EPSDT visits) may be worth the cost of those services (2-4).

We acknowledge study limitations. The SCRA linkage could not be made for 25.3% of the children who met the study’s other inclusion criteria; however, there were no significant differences between the 2 groups in their mean number of EPSDT visits. The AAP recommends an age-based schedule for EPSDT visits (18). The available data did not permit us to assess whether this schedule was followed. The results may be related to the timing of these visits as well as their number. Using the number of Medicaid claims to document clinical care use can underestimate the amount of service provided (33). Although the expected content of EPSDT visits is an accepted standard of care and is routinely available to physicians (14,21), specific services provided to children during these visits were not recorded in our data. Physician discretion may affect the content of these visits (34). If the services provided or the procedures for filing claims vary in a consistent fashion between the groups examined, the results could be biased. We have no reason to suspect systematic variation. In addition, because EPSDT visits do not typically occur during the first month of life, our calculation of the number of visits for the first year based on the 2nd through the 12th months of life is unlikely to have introduced bias (21).

Children in this analysis were not randomly selected. We addressed potential selection bias by adjusting for likely confounding variables. Nevertheless, the study design does not permit inferences about causality. Children with conditions that might predispose their parents to use more EPSDT services, such as physical anomalies at birth, not being full term and appropriately grown at birth, or having an extended newborn hospitalization, were excluded. Inferences from the results to children in these high-risk groups are not warranted.

Although regression models adjusted for several potential confounding variables, there was no direct control for parenting skills. The analysis controlled for various factors that may be associated with parenting skills, including maternal age and education, marital status, and family income (5,7,10,12,23). EPSDT use may be correlated with parenting skills, which may be more important determinants of school readiness than EPSDT visits. However, many parenting skills can be taught and are typically addressed by parental anticipatory guidance in well-child care (5,11). 

The data used for this study provided detailed information about mothers and infants and supported a linkage with education data, providing information that is rarely available for research in this area. Lack of objective evidence confirming the value of well-child visits has been a serious gap in available information. Lacking such evidence, some policy makers are uncertain whether increasing compliance with existing recommendations for well-child care in infancy through more provider outreach and parental education is worth any short-term added cost. This study provided evidence that well-child care during the first 2 years of life may contribute to school readiness for children insured by Medicaid. Pending additional research that may support this study’s findings that adherence to the recommended number of well-child visits may serve as an early marker for school readiness, the findings could be used to identify children who might benefit from early childhood programs designed to improve school readiness.

## Appendix. Domains and Skills Evaluated by the South Carolina Readiness Assessment

South Carolina kindergarten teachers conduct this assessment at the end of the kindergarten year to assess student readiness for first grade (25).

**I. Personal and Social Development**

**A. Self concept:** demonstrates self-confidence and shows initiative and self-direction.

**B. Self control:** follows classroom rules and routines, uses classroom materials purposefully and respectfully, and manages transitions and adapts to changes in routine.

**C. Approaches to learning:** shows eagerness and curiosity as a learner; sustains attention to a task, persisting even after encountering difficulty; and approaches tasks with flexibility and inventiveness.

**D. Interaction with others:** interacts easily with 1 or more children, interacts easily with familiar adults, participates in the group life of the class, and shows empathy and caring for others.

**E. Social problem solving:** seeks adult help and begins to use simple strategies to resolve conflicts.

**II. English Language Arts**

**A. Communication:** gains meaning by listening, follows directions that involve a series of actions, speaks clearly and conveys ideas effectively, and uses expanded vocabulary and language for a variety of purposes.

**B. Reading:** shows interest in and knowledge about books and reading; shows some understanding of concepts about print; demonstrates beginning phonemic awareness; knows letters and sounds and how they form words; and comprehends and responds to fiction and nonfiction text.

**C. Writing:** represents stories through pictures, dictation, and play; uses letter-like shapes, symbols, letters, and words to convey meaning; and understands purposes for writing.

**III. Mathematics**

**A. Mathematical processes:** uses and explains strategies to solve mathematical problems and uses words and representations to describe mathematical ideas.

**B. Numbers and operations:** shows understanding of number and quantity and shows emerging understanding of relationships between quantities.

**C. Patterns, relationships, and functions:** sorts objects into subgroups, classifying and comparing according to a rule; and recognizes, duplicates, and extends patterns.

**D. Geometry and spatial relations:** recognizes and describes some attributes of shapes and shows understanding of and uses direction, location, and position words.

**E. Measurement:** orders, compares, and describes objects by size, length, capacity, and weight; explores and uses common instruments for estimating and measuring during work or play; and shows awareness of time concepts.

**F. Data collection and probability:** collects data and makes records using lists or graphs.
